# Supplementary material for: A triple-defense electrocatalyst for robust seawater oxidation
Source: Nat Commun. 2025 Nov 24;16:10327. doi: 10.1038/s41467-025-65272-3 (PMC12644857; doi:10.1038/s41467-025-65272-3)
Supplement: Supplementary file 1 — Supplementary Information [file 41467_2025_65272_MOESM1_ESM.pdf]

Supplementary Information

A triple-defense electrocatalyst for robust seawater oxidation

Zixiao Li[1,2,#], Jie Liang[2,#], Shaohuan Hong[3,#], Yuchun Ren[1], Min Zhang[1], Shengjun Sun[1], Zhengwei Cai[1], Chaoxin Yang[1], Hefeng Wang[1], Yongsong Luo[4], Shanhu Liu[5], Yongchao Yao[6*], Feng Gong[3*], Xuping Sun[1,4*] & Bo Tang[1,7*]

[1]College of Chemistry, Chemical Engineering and Materials Science, Shandong Normal University, Jinan 250014, Shandong, China. [2]Institute of Fundamental and Frontier Sciences, University of Electronic Science and Technology of China, Chengdu 610054, Sichuan, China. [3]MOE Key Laboratory of Energy Thermal Conversion and Control, School of Energy and Environment, Southeast University, Nanjing 211189, Jiangsu, China. [4]Center for High Altitude Medicine, West China Hospital, Sichuan University, Chengdu 610041, Sichuan, China. [5]College of Chemistry and Molecular Sciences, Henan University, Kaifeng 475004, Henan, China. [6]Department of Laboratory Medicine, Precision Medicine Center, West China Hospital, Sichuan University, Chengdu 610041, Sichuan, China. [7]Laoshan Laboratory, Qingdao 266237, Shandong, China. [#]These contributed equally to this work.

[*]Correspondence and requests for materials should be addressed to Y.Y. (email: hatuu@wchscu.edu.cn) or F.G. (email: gongfeng@seu.edu.cn) or B.T. (email: tangb@sdnu.edu.cn) or X.S. (email: xpsun@uestc.edu.cn)

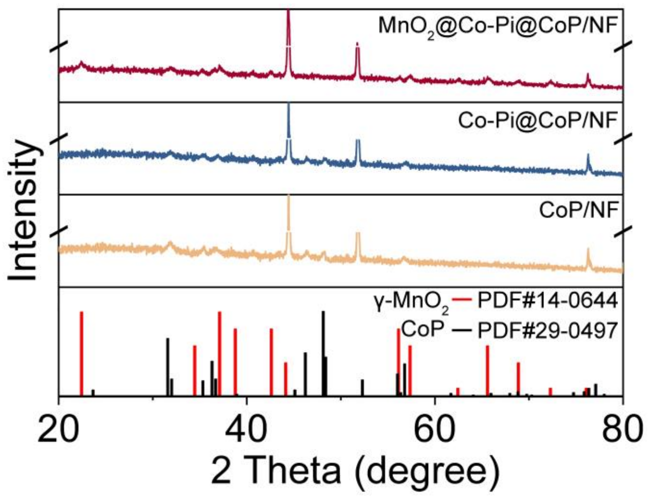

Supplementary Fig. 1 | XRD patterns of CoP/NF, Co-Pi@CoP/NF, and MnO_2@Co-Pi@CoP/NF. Source data are provided as a Source Data file.

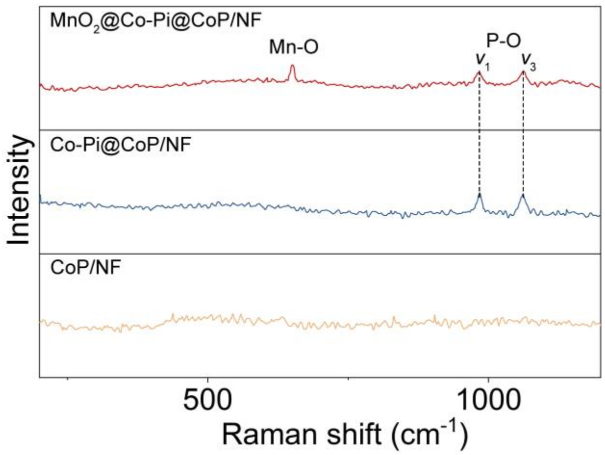

Supplementary Fig. 2 | Raman spectra of CoP/NF, Co-Pi@CoP/NF, and MnO_2@Co-Pi@CoP/NF. Source data are provided as a Source Data file.

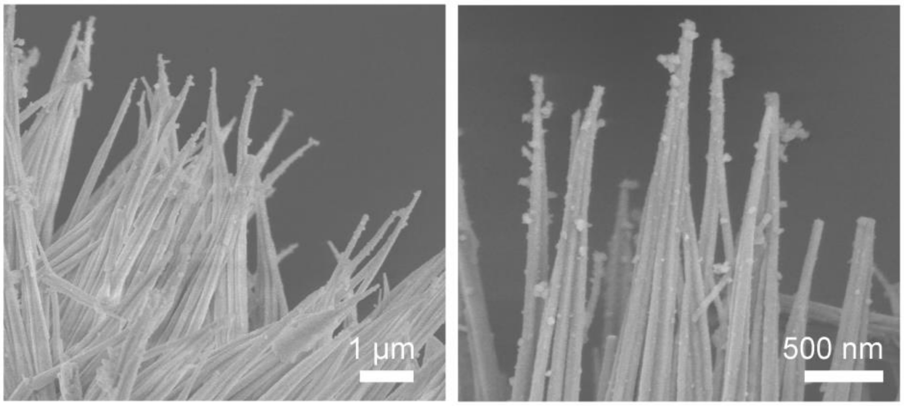

Supplementary Fig. 3 | SEM images for Co-Pi@CoP/NF.

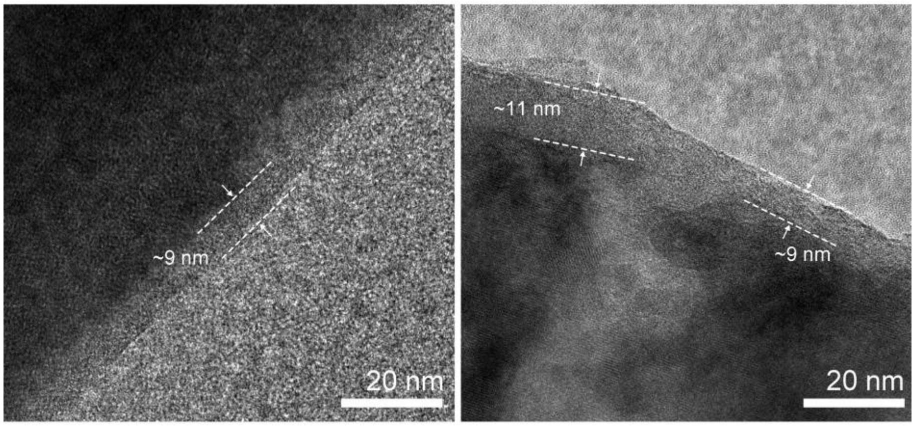

Supplementary Fig. 4 | HRTEM images of MnO_2@Co-Pi@CoP at two different areas.

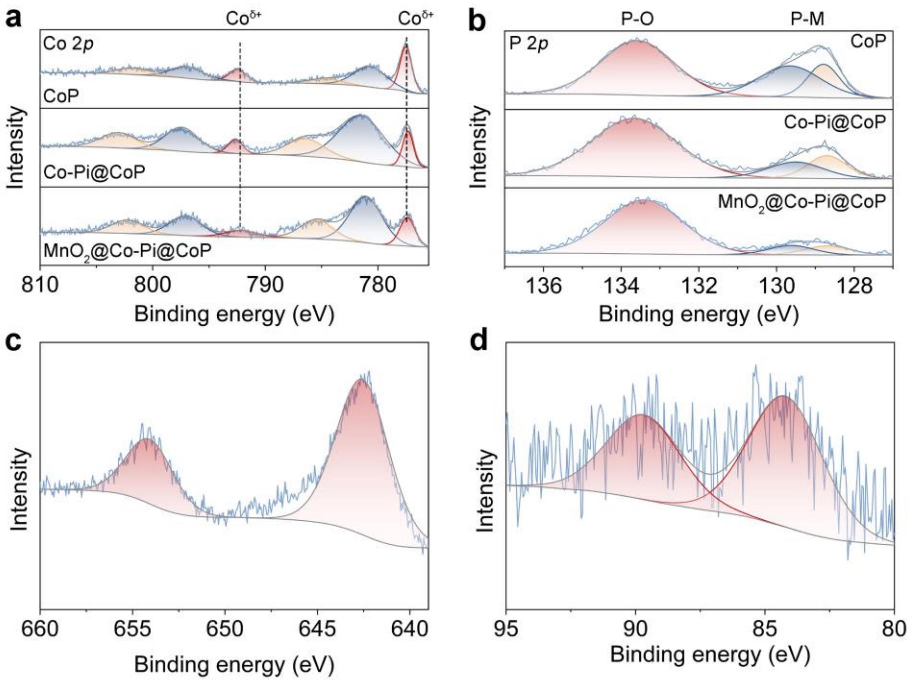

Supplementary Fig. 5 | High-resolution XPS spectra. (a) XPS spectra of CoP, Co-Pi@CoP, and MnO_2@Co-Pi@CoP in Co 2*p* region. (b) XPS spectra of CoP, Co-Pi@CoP, and MnO_2@Co-Pi@CoP in P 2*p* region. (c) XPS spectrum of MnO_2@Co-Pi@CoP in Mn 2*p* region. (d) XPS spectrum of MnO_2@Co-Pi@CoP in Mn 3*s* region. Source data are provided as a Source Data file.

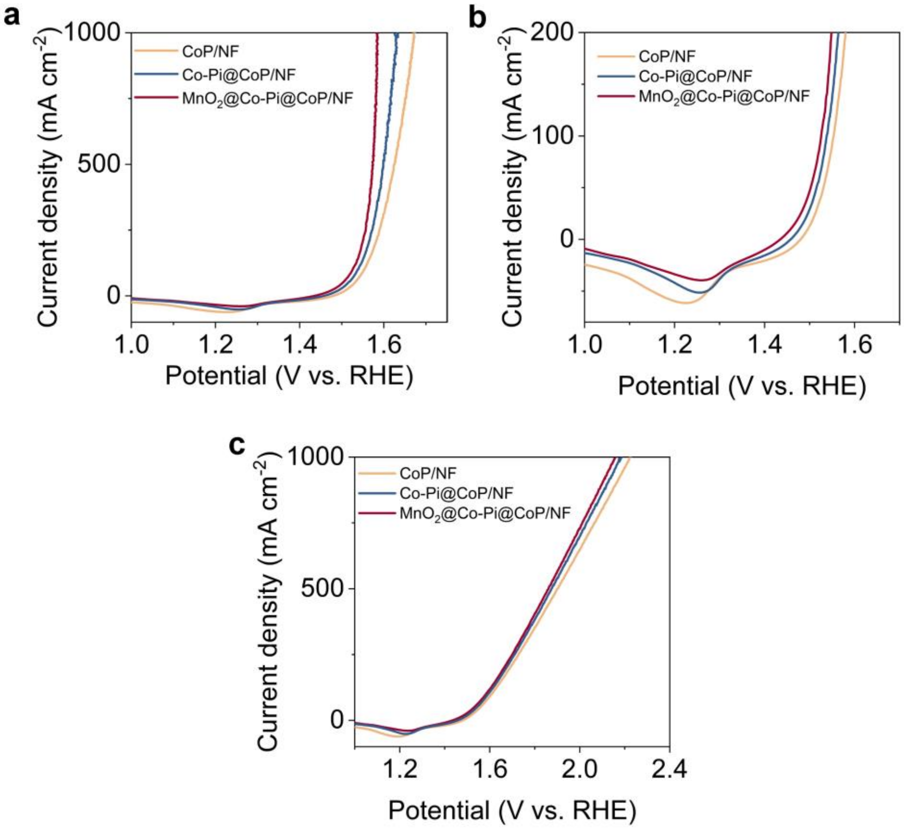

Supplementary Fig. 6 | LSV measurements. (a) LSV curves of CoP/NF, Co-Pi@CoP/NF, and MnO_2@Co-Pi@CoP/NF with 85% iR correction. (b) LSV curves with 85% iR correction at a low-*j* region. (c) LSV curves without iR correction. Source data are provided as a Source Data file.

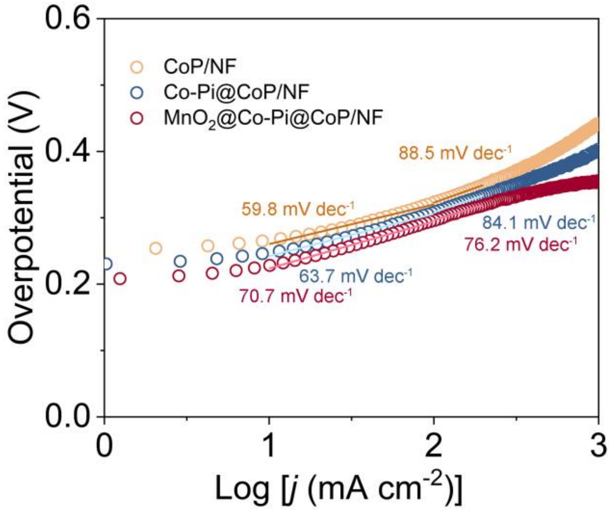

Supplementary Fig. 7 | Tafel values of CoP/NF, Co-Pi@CoP/NF, and MnO_2@Co-Pi@CoP/NF at *j* of 10-100 and 100-200 mA cm^{-2} regions. Source data are provided as a Source Data file.

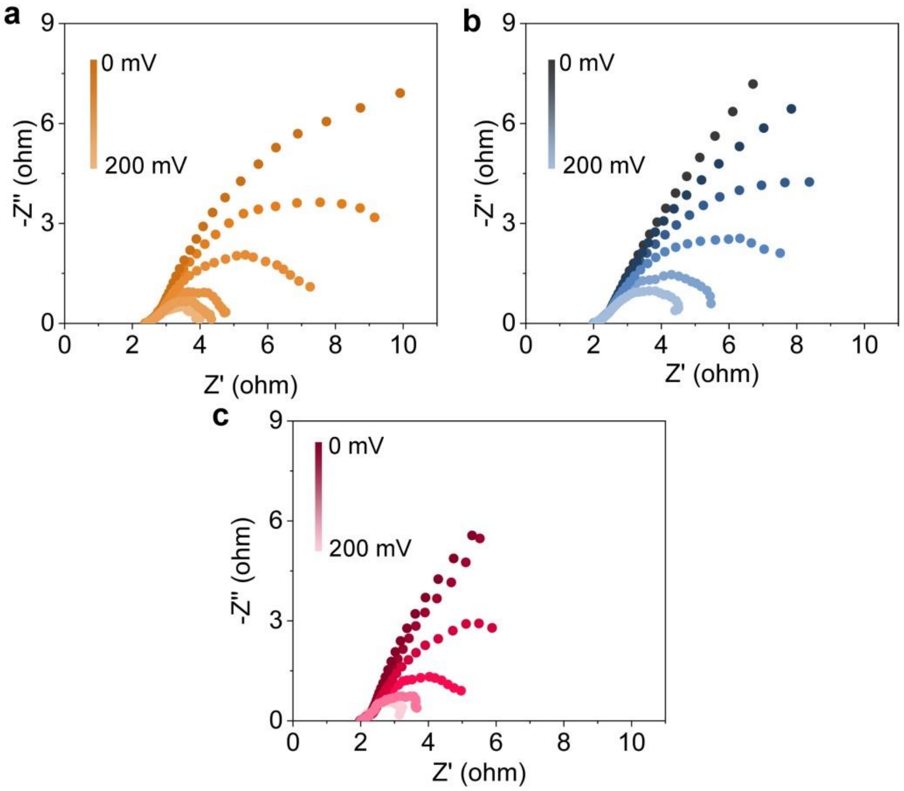

Supplementary Fig. 8 | Nyquist plots. Nyquist plots of (a) CoP/NF, (b) Co-Pi@CoP/NF, and (c) MnO_2@Co-Pi@CoP/NF at an overpotential range of 0-200 mV. Source data are provided as a Source Data file.

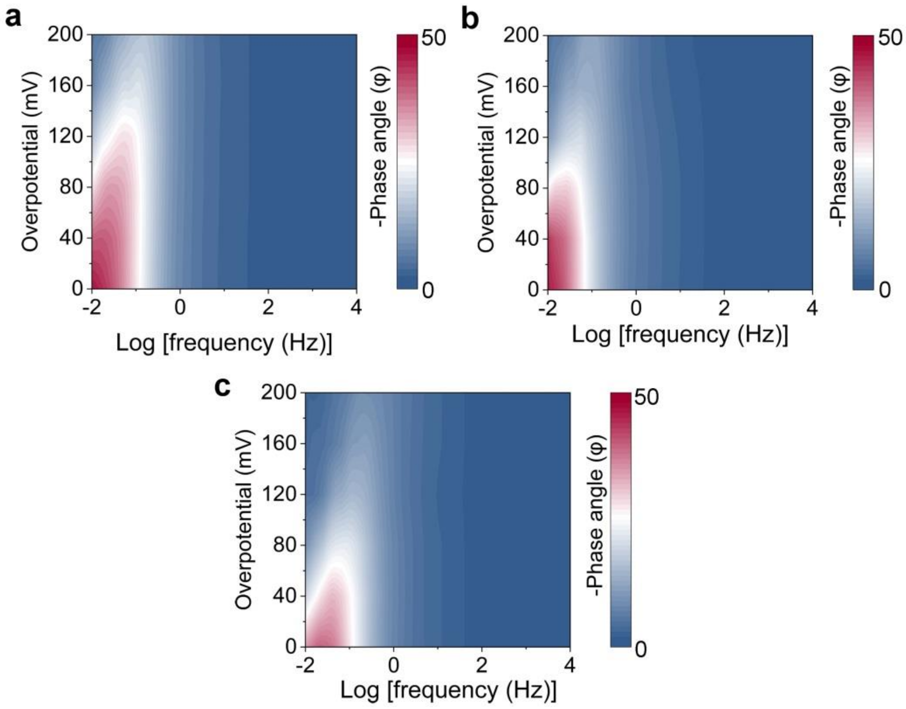

Supplementary Fig. 9 | Bode plots. Bode plots of (a) CoP/NF, (b) Co-Pi@CoP/NF, and (c) MnO_2@Co-Pi@CoP/NF at an overpotential range of 0-200 mV. Source data are provided as a Source Data file.

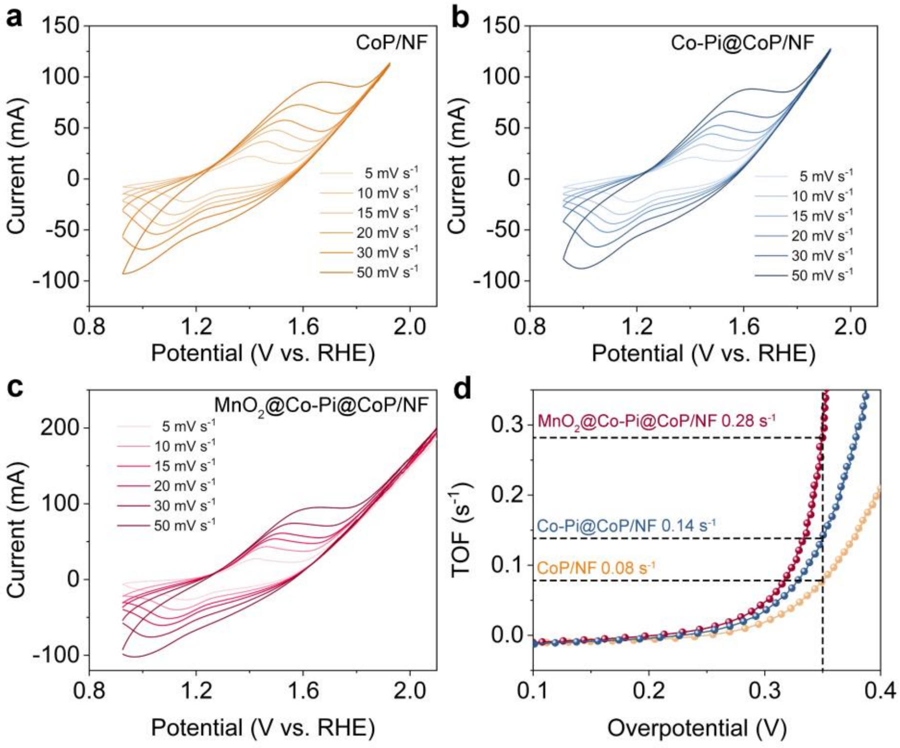

Supplementary Fig. 10 | CV curves and the TOF values. (a-c) CV curves of CoP/NF, Co-Pi@CoP/NF, and MnO_2@Co-Pi@CoP/NF. (d) The corresponding TOF curves. Source data are provided as a Source Data file.

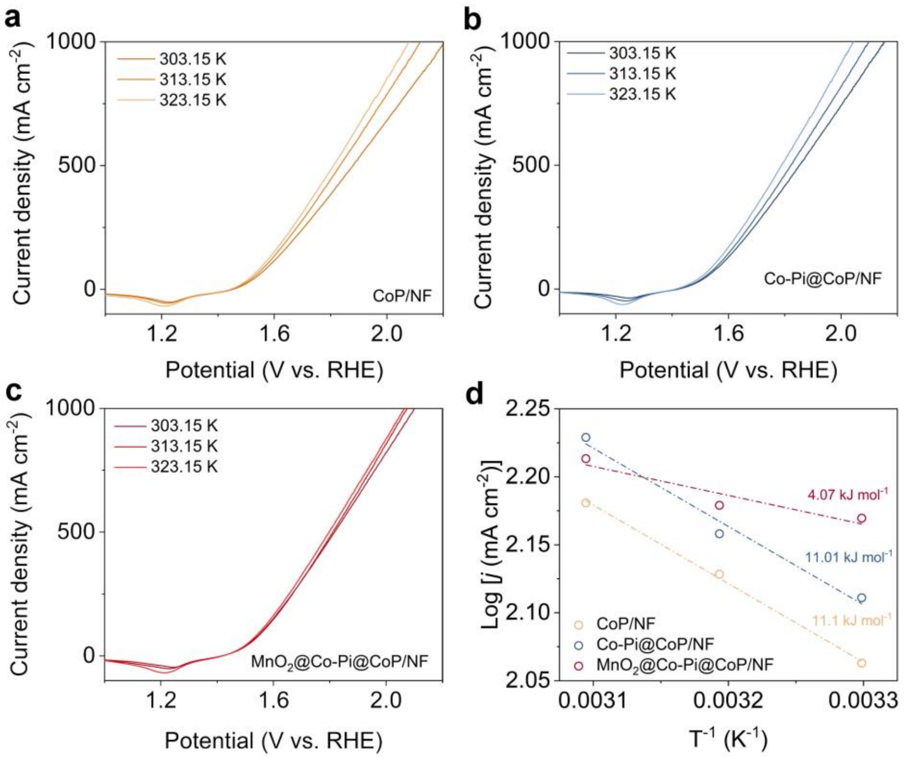

Supplementary Fig. 11 | Activation energy. (a-c) Polarization curves of CoP/NF, Co-Pi@CoP/NF, and MnO_2@Co-Pi@CoP/NF in alkaline seawater with different temperatures. (d) Arrhenius plots of the kinetic currents at 1.6 V vs. RHE. Source data are provided as a Source Data file.

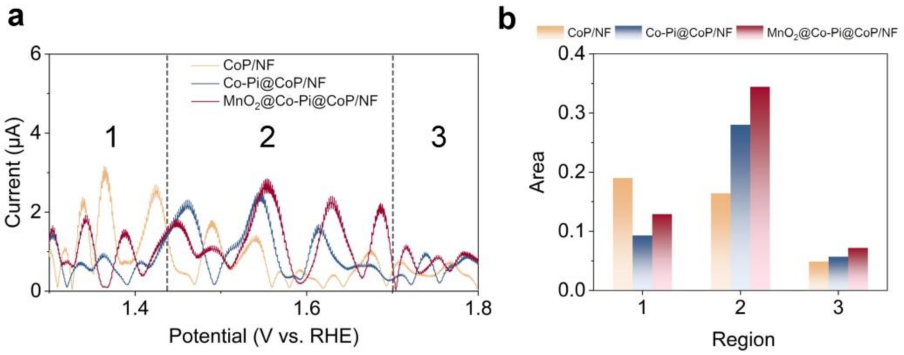

Supplementary Fig. 12 | FtacV measurements of the samples. (a) FtacV curves of CoP/NF, Co-Pi@CoP/NF, and MnO_2@Co-Pi@CoP/NF, and (b) corresponding regions area. Source data are provided as a Source Data file.

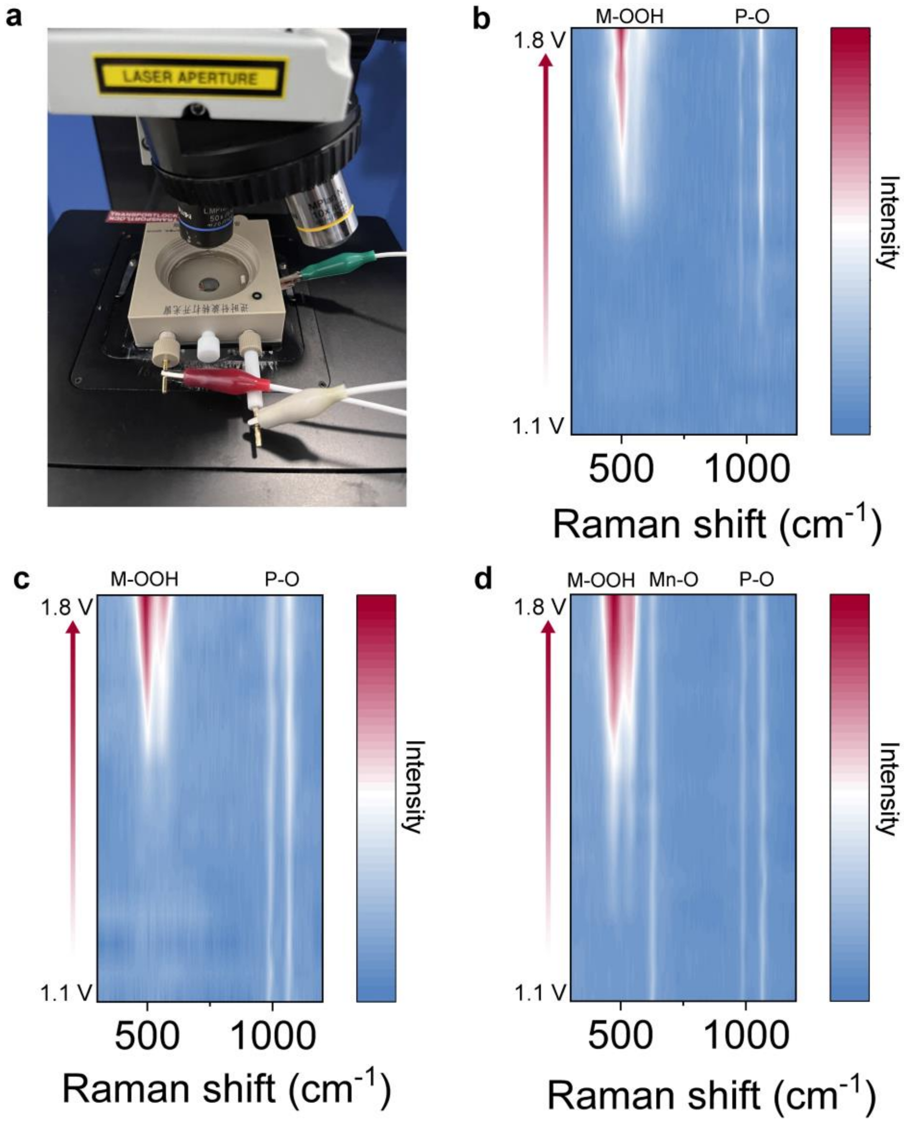

Supplementary Fig. 13 | In situ Raman spectra of electrodes in alkaline seawater. (a) Photograph of an electrochemical cell for in situ Raman test. (b) CoP/NF. (c) Co-Pi@CoP/NF. (d) MnO_2@Co-Pi@CoP/NF. Source data are provided as a Source Data file.

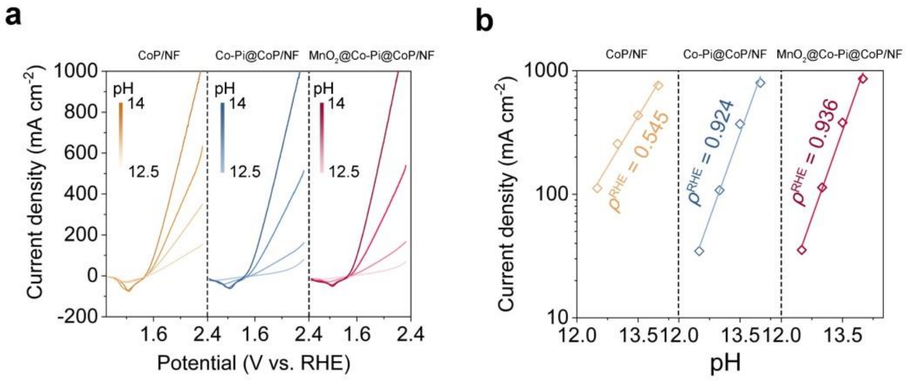

Supplementary Fig. 14 | pH dependent electrochemical experiments. (a) Polarization curves (without iR correction) and (b) ρ^{RHE} values of CoP/NF, Co-Pi@CoP/NF, and MnO_2@Co-Pi@CoP/NF. Source data are provided as a Source Data file.

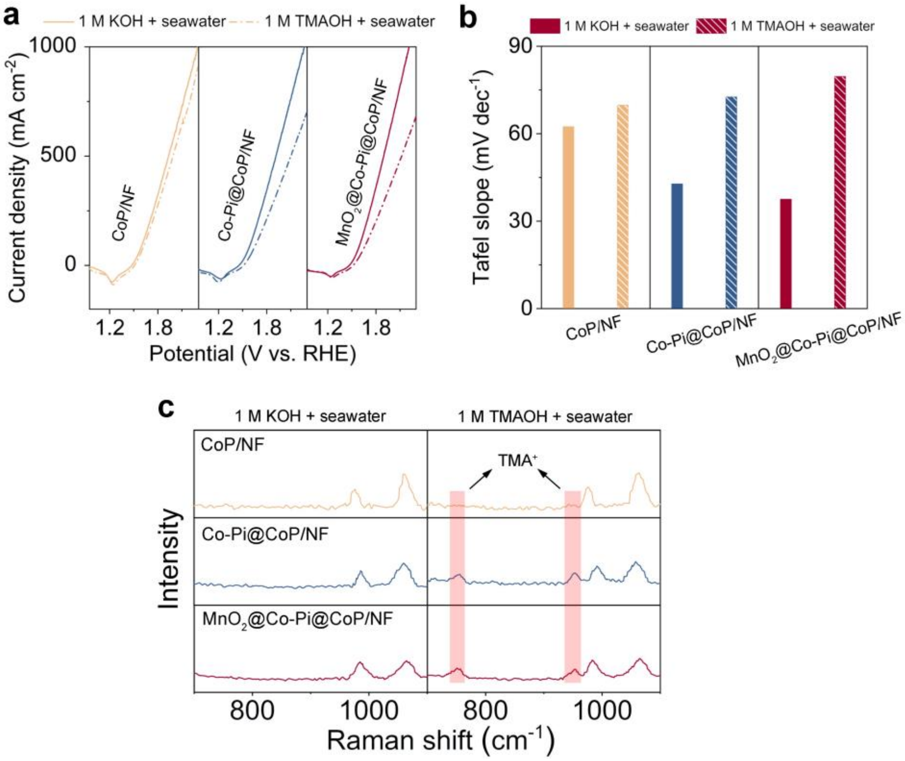

Supplementary Fig. 15 | Evidence for the LOM pathway. (a,b) Polarization curves and corresponding Tafel values of CoP/NF, Co-Pi@CoP/NF, and MnO_2@Co-Pi@CoP/NF in the electrolyte with or without TMAOH. (c) Raman spectra of various catalysts tested after testing with or without TMAOH. Source data are provided as a Source Data file.

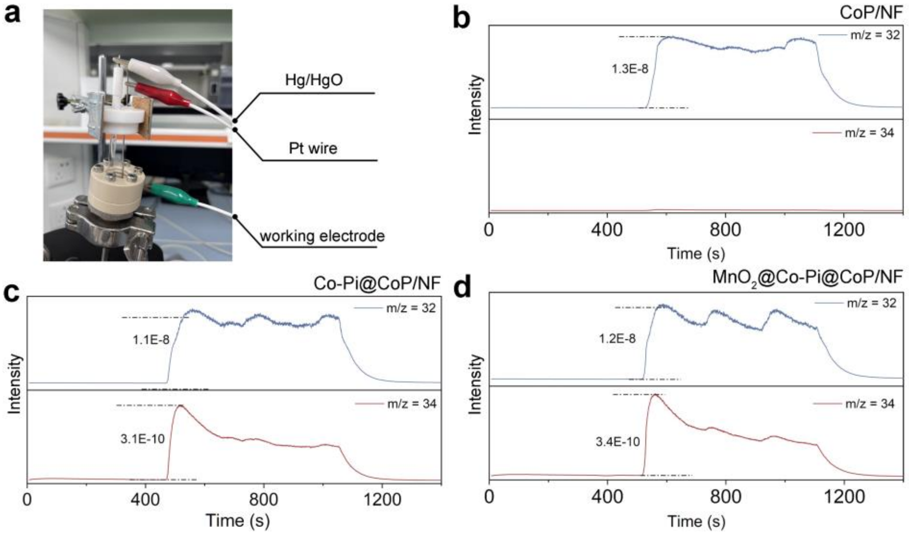

Supplementary Fig. 16 | Online DEMS spectra. (a) Photograph of an electrochemical cell for online DEMS test.Online mass spectrometry spectra of (b) CoP/NF, (c) Co-Pi@CoP/NF, and (d) MnO_2@Co-Pi@CoP/NF. Source data are provided as a Source Data file.

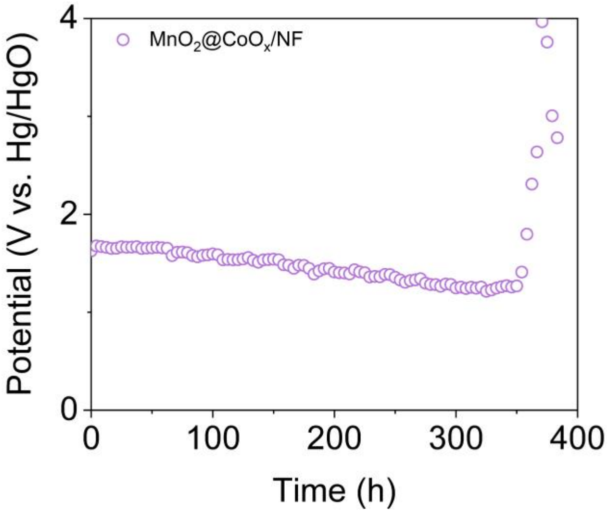

Supplementary Fig. 17 | Lifespans of MnO_2@CoO_x/NF at 2 A cm^{-2} in alkaline seawater. Source data are provided as a Source Data file.

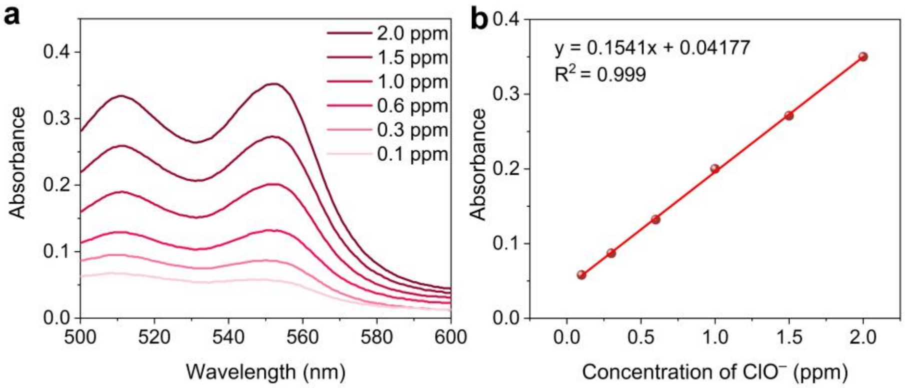

Supplementary Fig. 18 | UV-vis absorption spectra and calibration curve. (a) UV-vis absorption spectra of various active chlorine concentrations. (b) Calibration curve for evaluating ClO^- concentrations of the electrolyte. Source data are provided as a Source Data file.

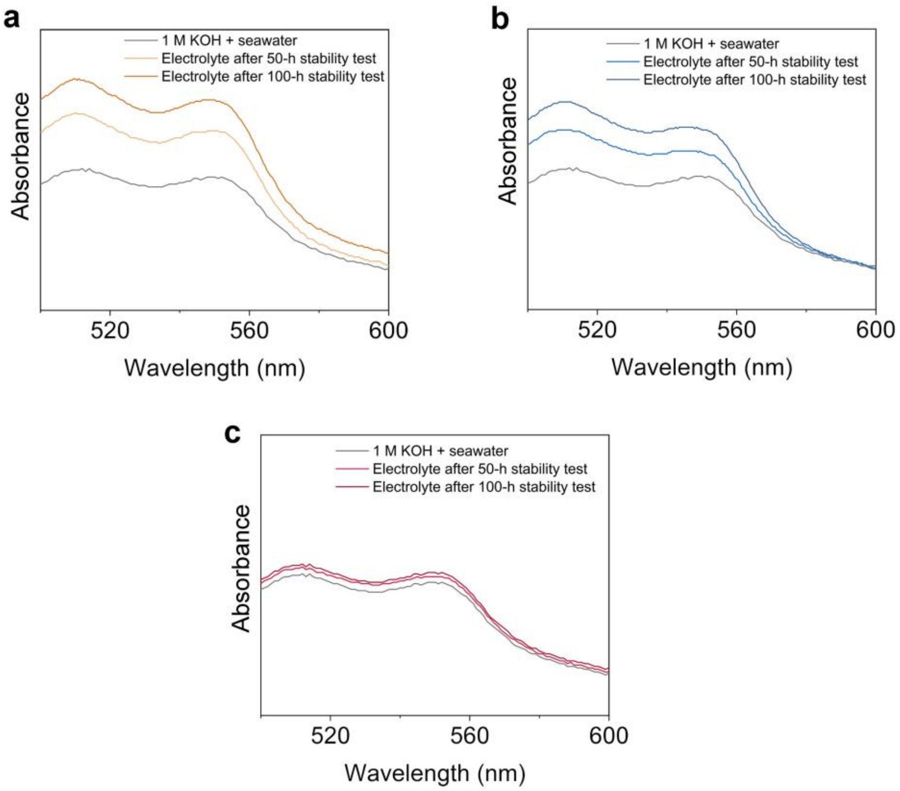

Supplementary Fig. 19 | UV-vis absorption spectra of electrolytes from (a) CoP/NF, (b) Co-Pi@CoP/NF, and (c) MnO_2@Co-Pi@CoP/NF after tests at 1 A cm^{-2}. Source data are provided as a Source Data file.

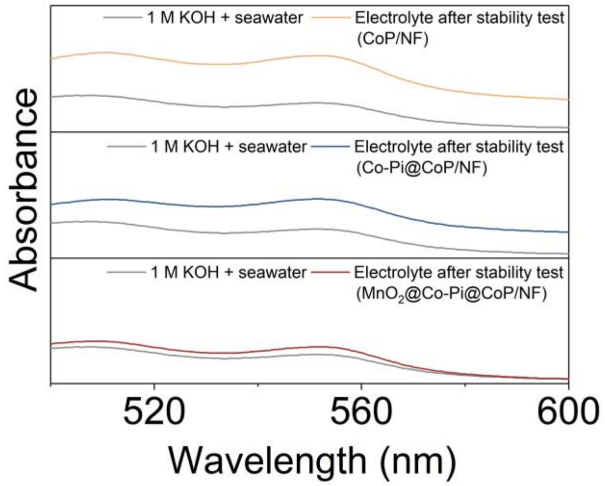

Supplementary Fig. 20 | UV-vis absorption spectra of electrolytes from CoP/NF, Co-Pi@CoP/NF, and MnO_2@Co-Pi@CoP/NF after stability tests at 2 A cm^{-2}. Source data are provided as a Source Data file.

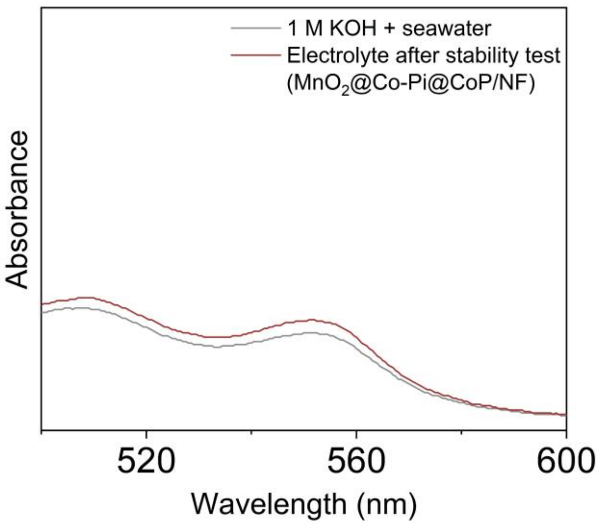

Supplementary Fig. 21 | UV-vis absorption spectra of electrolytes from MnO_2@Co-Pi@CoP/NF after stability tests at 1 A cm^{-2}. Source data are provided as a Source Data file.

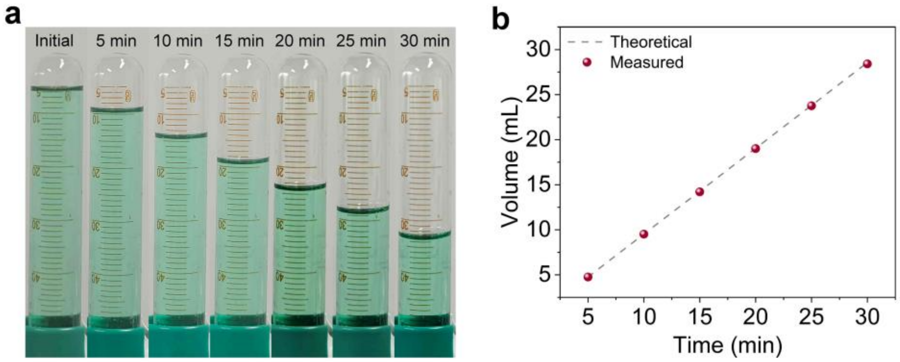

Supplementary Fig. 22 | (a) Digital photographs of the collected O_2 and (b) comparison between the collected and theoretical O_2 amount for MnO_2@Co-Pi@CoP/NF at a *j* of 1000 mA cm^{-2} in alkaline seawater. Source data are provided as a Source Data file.

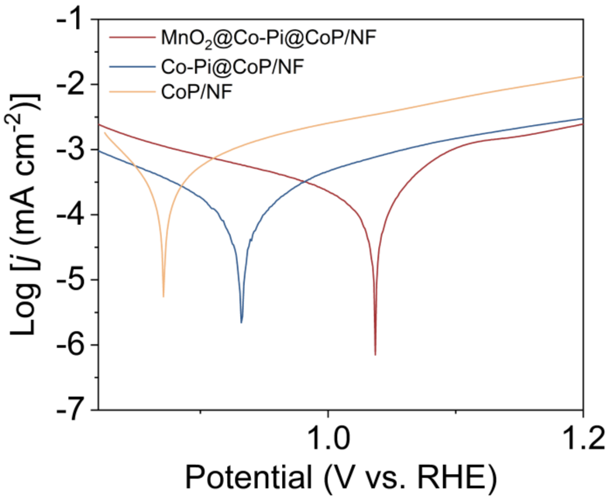

Supplementary Fig. 23 | Polarization curve-based Tafel plots of CoP/NF, Co-Pi@CoP/NF, and MnO_2@Co-Pi@CoP/NF. Source data are provided as a Source Data file.

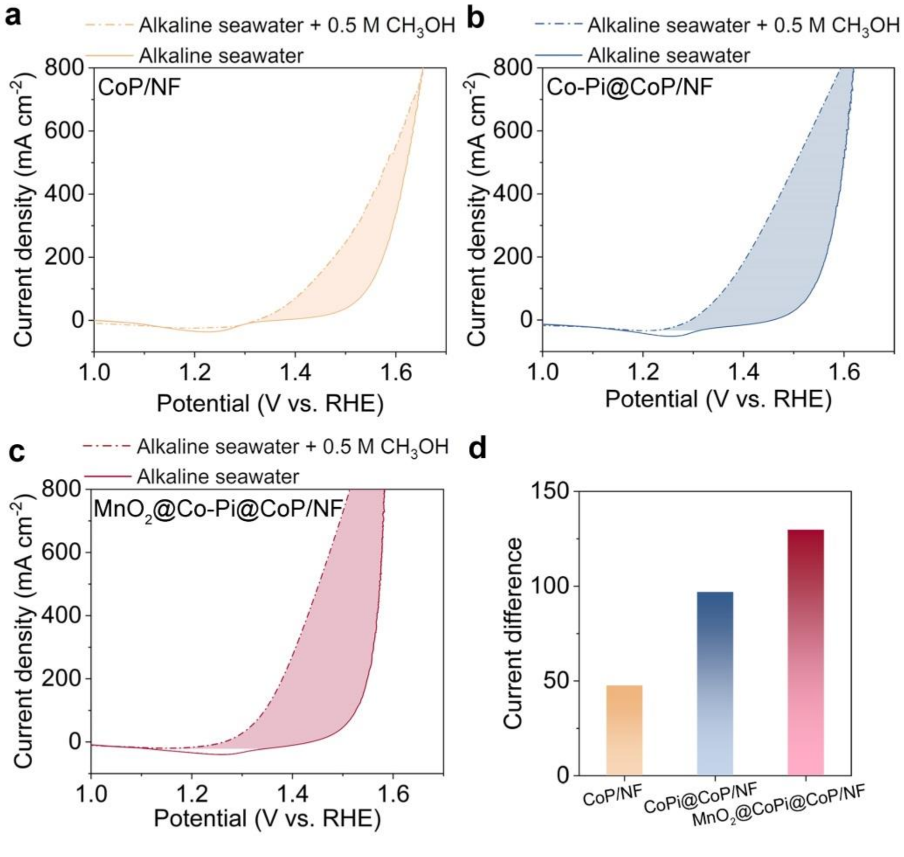

Supplementary Fig. 24 | Methanol oxidation reaction. (a-c) Polarization curves of CoP/NF, Co-Pi@CoP/NF, and MnO_2@Co-Pi@CoP/NF in alkaline seawater with (dashed lines) and without (solid lines) 1 M methanol. (d) The corresponding current difference between the polarization curves. Enhanced *OH adsorption abilities can be validated experimentally using methanol as a molecular probe. Methanol oxidation reaction (MOR) operates through a well-established mechanism, where methanol molecules typically nucleophilically attack the electrophilic *OH. Therefore, a strengthened *OH-adsorption electrode will show high activity in MOR. Source data are provided as a Source Data file.

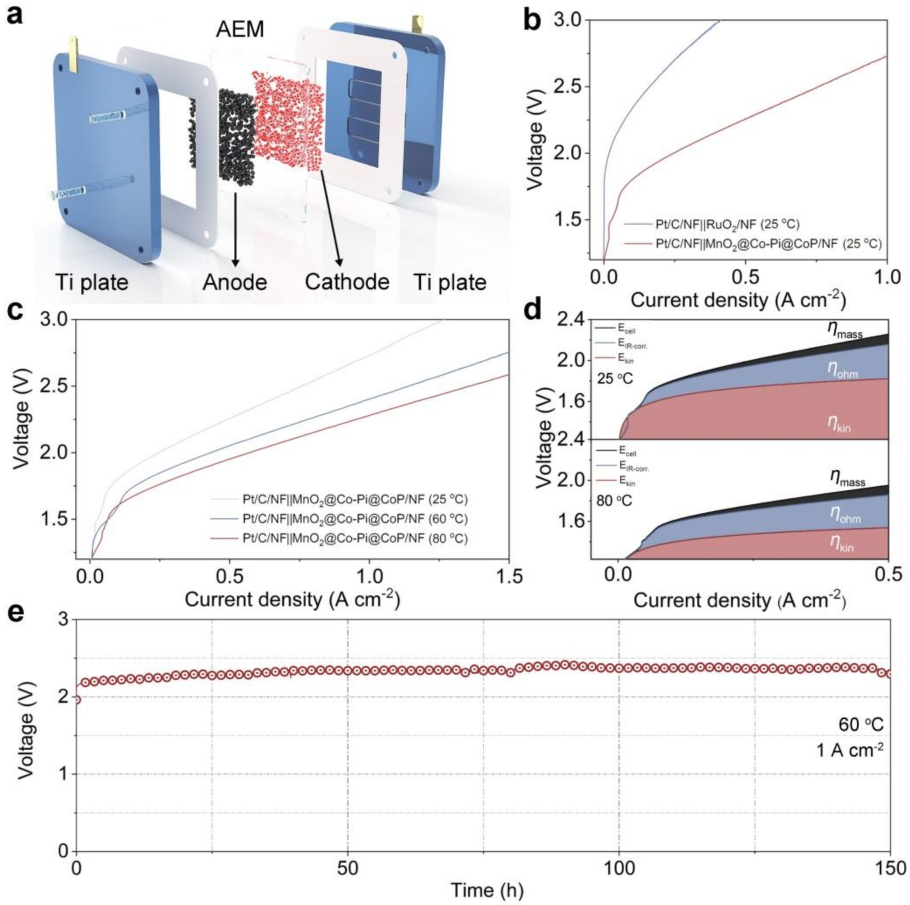

Supplementary Fig. 25 | Electrochemical performance of the anion exchange membrane electrolyzer. (a) Schematic diagram of the seawater splitting device. (b) Polarization curves recored for Pt/C/NF||RuO_2/NF and Pt/C/NF||MnO_2@Co-Pi@CoP/NF. (c) Polarization curves recorded for Pt/C/NF||MnO_2@Co-Pi@CoP/NF at 25, 60, and 80 °C. (d) Polarization curve subdivision results. (e) Durability test for MnO_2@Co-Pi@CoP/NF-based anion exchange membrane electrolyzer at 1 A cm^{-2}. Source data are provided as a Source Data file.

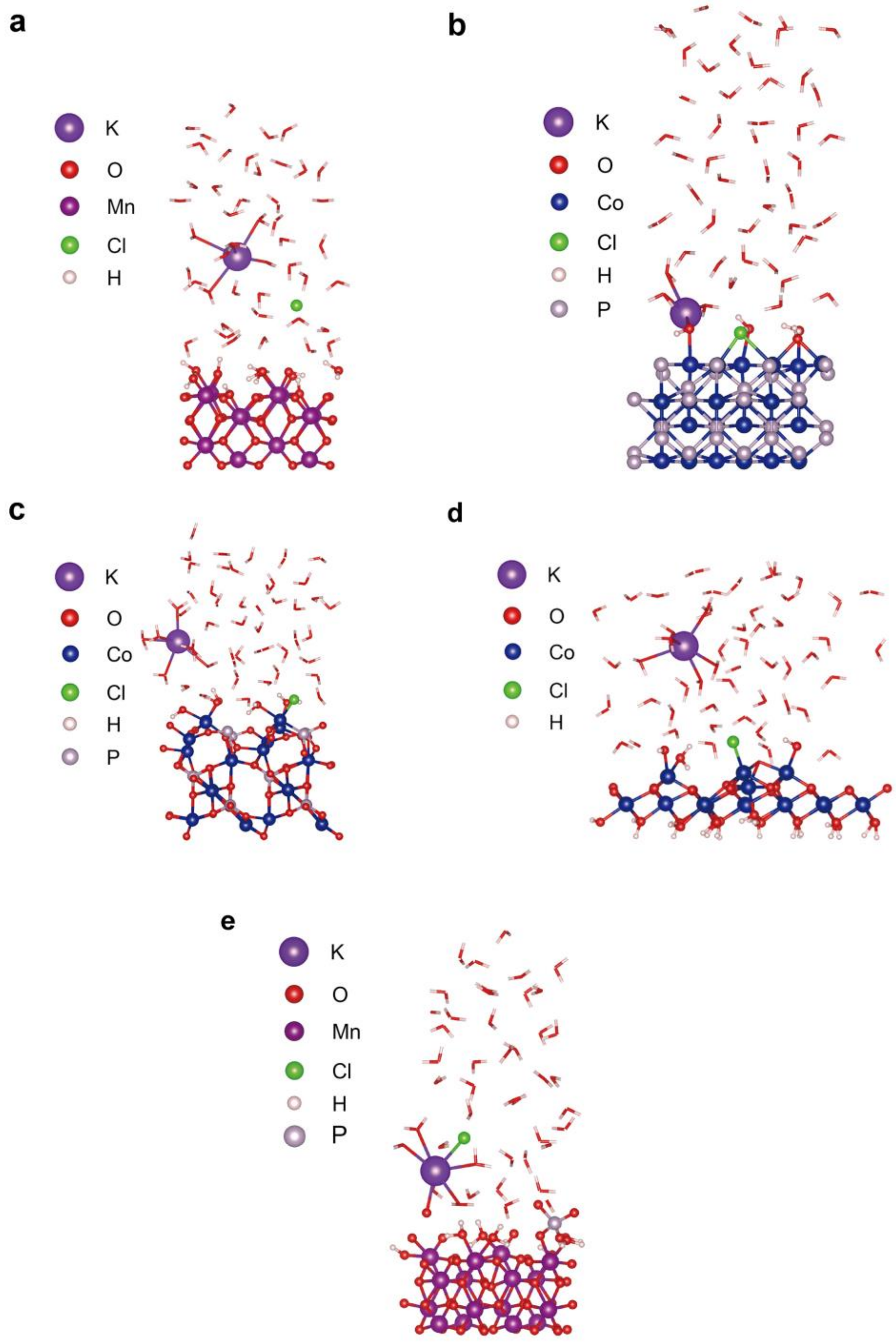

Supplementary Fig. 26 | AIMD simulations showing the configuration of the electrolyte system above the electrode surfaces. (a) Schematic diagram of explicit solvent model of γ-MnO_2 (100). (b) Schematic diagram of explicit solvent model of CoP (011). (c) Schematic diagram of explicit solvent model of Co-Pi (001). (d) Schematic diagram of explicit solvent model of CoOOH (001). (e) Schematic diagram of explicit solvent model of PO_4^{3-}@γ-MnO_2 (100).

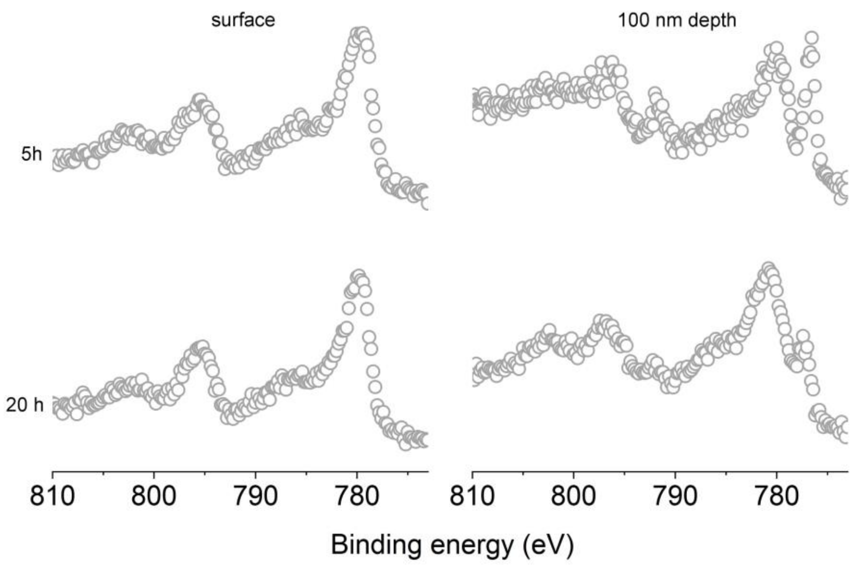

Supplementary Fig. 27 | Depth- and catalytic reaction time-dependent XPS spectra in Co 2*p* region. Source data are provided as a Source Data file.

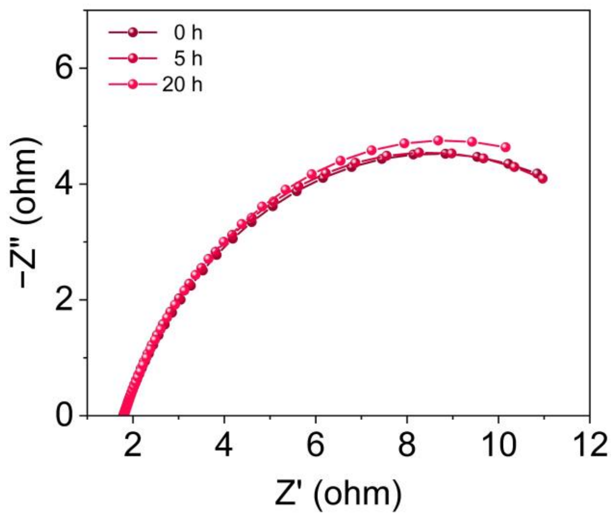

Supplementary Fig. 28 | Nyquist plots of MnO_2@Co-Pi@CoP/NF after different reaction time. Source data are provided as a Source Data file.

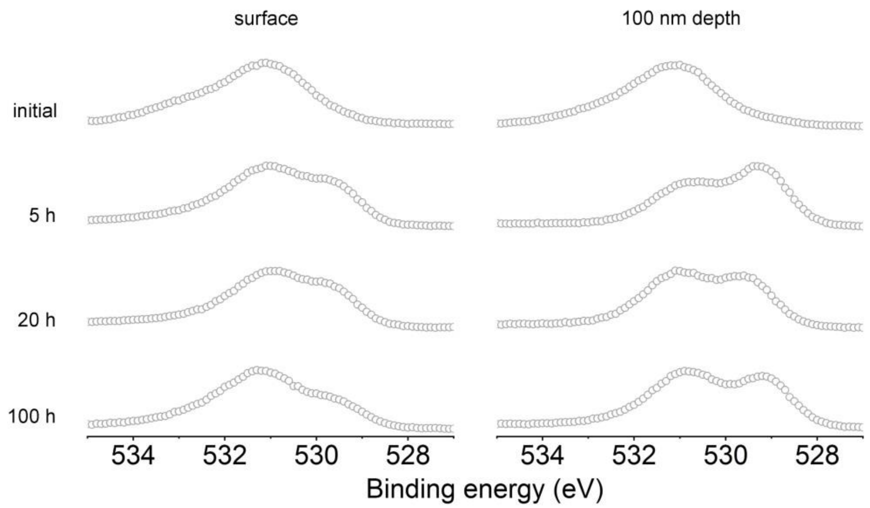

Supplementary Fig. 29 | Depth- and catalytic reaction time-dependent XPS spectra in O 1*s* region. Source data are provided as a Source Data file.

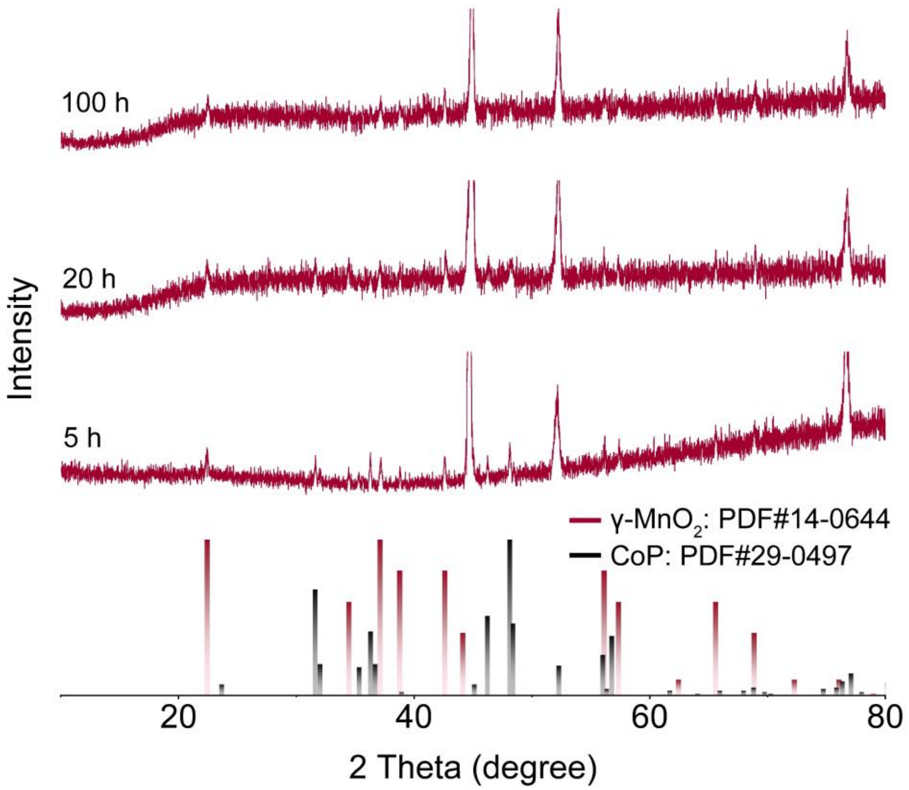

Supplementary Fig. 30 | XRD patterns of MnO_2@Co-Pi@CoP/NF after 5-h eASO, 20-h eASO, and 100-h eASO. Source data are provided as a Source Data file.

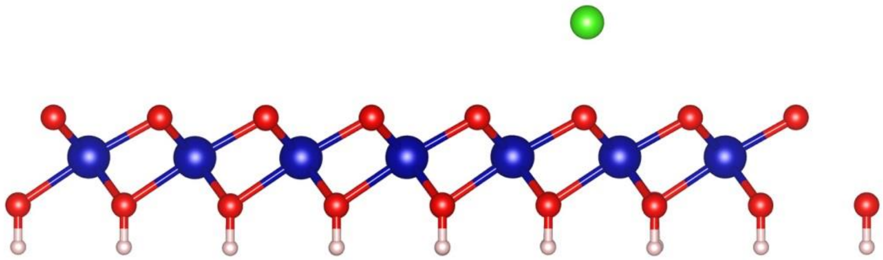

Supplementary Fig. 31 | Atomic atructure of Cl^{-} on CoOOH (001).

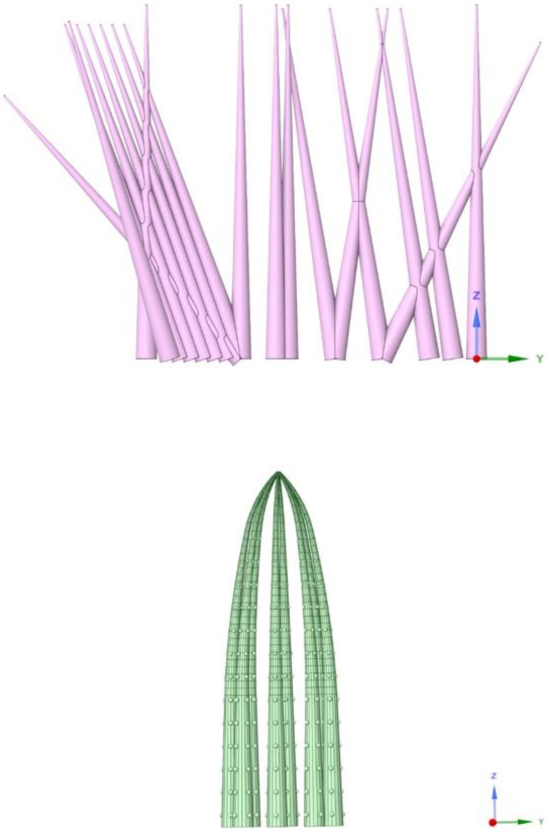

Supplementary Fig. 32 | Geometric modeling for the typical arrangements of nanowires and the special tip-connected arrangement of nanowires.

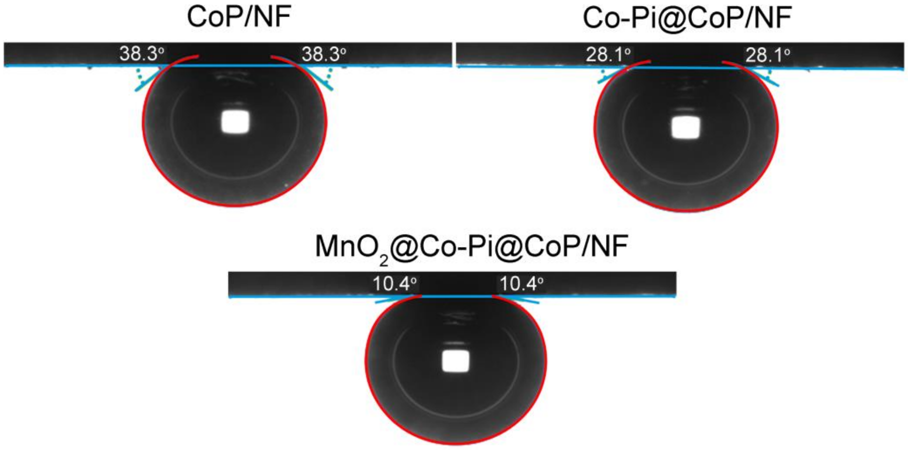

Supplementary Fig. 33 | Gas contact angles of CoP/NF, Co-Pi@CoP/NF, and MnO_2@Co-Pi@CoP/NF.

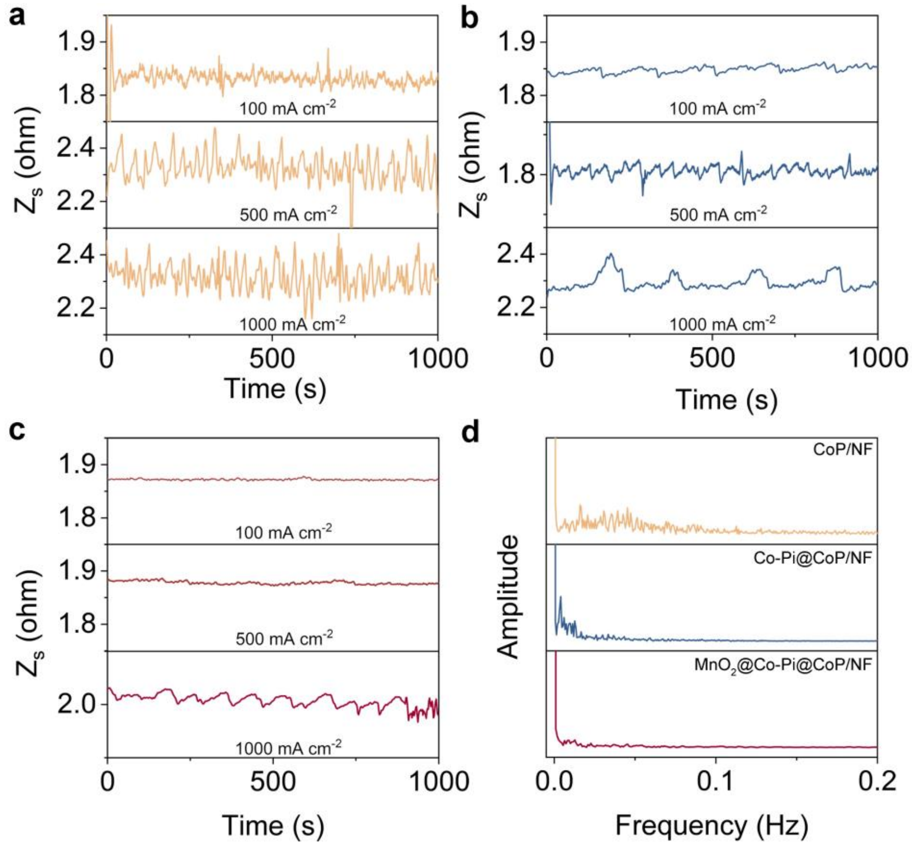

Supplementary Fig. 34 | Operando dynamic resistance variation. Dynamic resistance variation of (a) CoP/NF, (b) Co-Pi@CoP/NF, and (c) MnO_2@Co-Pi@CoP/NF at 100, 500, and 1000 mA cm^{-2}. (d) Fast Fourier transform curves of CoP/NF, Co-Pi@CoP/NF, and MnO_2@Co-Pi@CoP/NF at 1000 mA cm^{-2}. Source data are provided as a Source Data file.

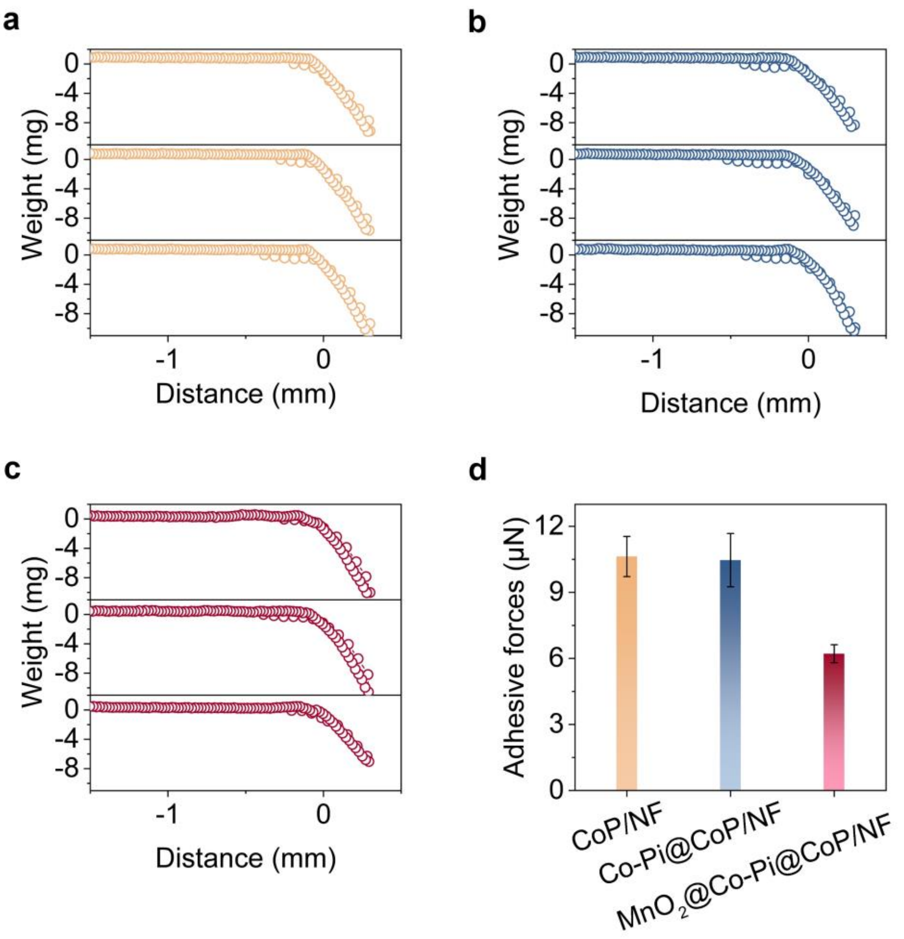

Supplementary Fig. 35 | Adhesive forces measurement. Adhesive forces curves of (**a**) CoP/NF, (**b**) Co-Pi@CoP/NF, and (**c**) MnO_2@Co-Pi@CoP/NF. (**d**) The corresponding adhesive forces data. Source data are provided as a Source Data file.

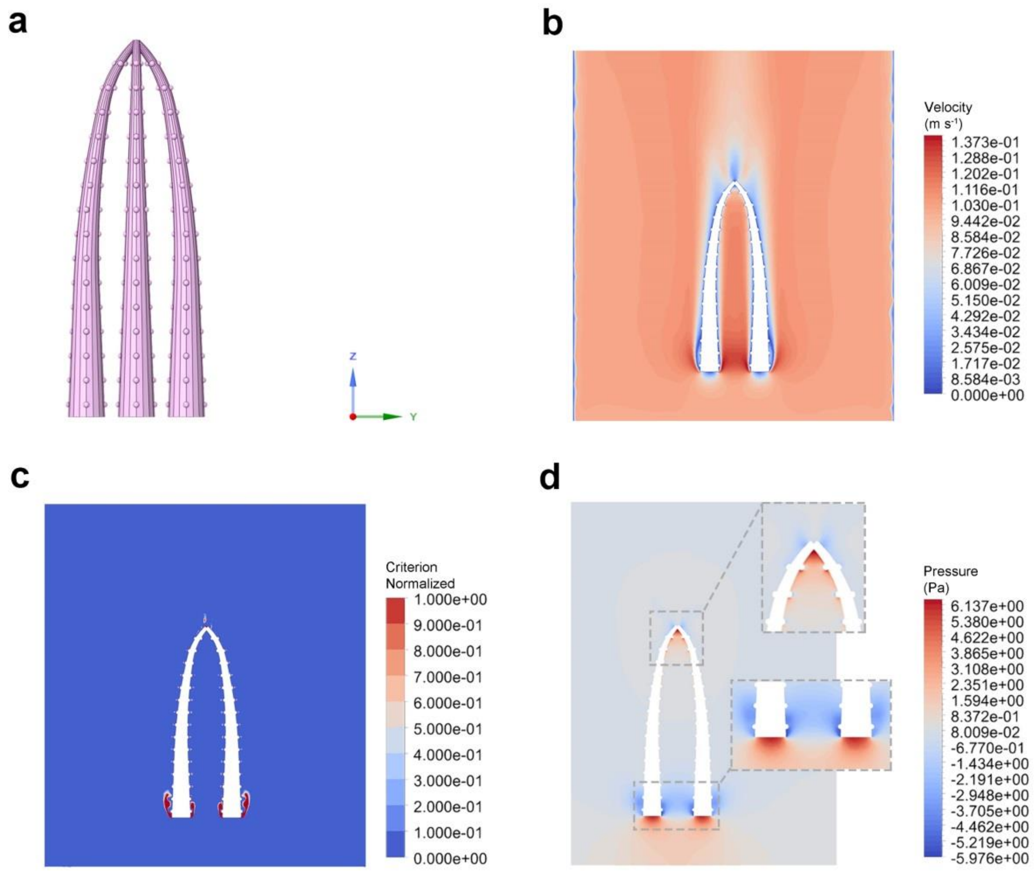

Supplementary Fig. 36 | Simulations of the velocity field, bubble evolution, and force distribution on another superior structure. a, Geometric modeling of the structure with different numbers of nanowires within the cage-like structure. **b**, Velocity field for the counterpart. **c**, Gas bubble distribution on the counterpart. **d**, Distribution of forces on the counterpart from the bubble.

Supplementary Table 1. Research efforts to enhance the OER electrode lifespan in the past few years. Details: electrolyte, *j*, time (t), mass loading (M), surface area (s), temperature (T), and test system (2 or 3 electrodes).

Catalyst	Electrolyte	*j* (mA cm^{-2})	t (h)	M (mg cm^{-2})	s (cm^{-2})	T (^oC)	Test system	Ref.
MnO_2@Co-Pi@CoP/NF	1 M KOH + seawater	1000	3000	~2.7	0.25	~25 ^oC	3	This work
Fe-NiSOH/NF	1 M KOH + seawater	500	900	-	-	-	3	*Energy Environ. Sci.* **15**, 4647–4658 (2022)
BZ-NiFe-LDH/NF	1 M KOH + seawater	500	100	-	0.25	~25 ^oC	3	*Nano Res. Energy* **1**, e9120028 (2022)
$(NiFe)C_2O_4$/NF	1 M KOH + seawater	1000	600	-	0.25	~25 ^oC	3	*Angew. Chem. Int. Ed.* **63**, e202316522 (2024)
CrO_4^{2-}-NiFe LDH/Cr_2O_3/NF	1 M KOH + seawater	1000	1000	~2.0	0.25	~25 ^oC	3	*Nat. Commun.* **15**, 6624 (2024)
$NiMoS_x$@NiFe-LDH/NF	1 M KOH + seawater	500	500	-	0.25	-	3	*Inorg. Chem. Front.* **10**, 2766–2775 (2023)
NiIr-LDH/NF	1 M KOH + seawater	500	650	1.0	1	-	2	*J. Am. Chem. Soc.* **144**, 9254–9263 (2022)
RuMoNi/NF	1 M KOH + seawater	500	3000	3.6	1	20 ± 2 ^oC	3	*Nat. Commun.* **14**, 3607 (2023)
MoO_3@CoO/CC	1 M KOH + seawater	600	1000	-	-	~25 ^oC	3	*Nat. Commun.* **15**, 2481 (2024)

Ag/NiFe LDH/NF	1 M KOH + seawater	1000	1000	-	-	-	3	*Nano Energy* **98**, 107212 (2022)
CoFePBA/Co_2P/NF	1 M KOH + seawater	1000	1000	-	-	~25 ^oC	3	*Angew. Chem. Int. Ed.* **62**, e202309882 (2023)
NiFe-LDH@Ag/NF	1 M KOH + seawater	400	2500	-	1	~25 ^oC	2	*Adv. Mater.* **36**, 2306062 (2024)
CoFe-Ci@GQDs/NF	1 M KOH + 0.5 M NaCl	1250	2800	-	-	~25 ^oC	3	*Nat. Sustain.* **7**, 158–167 (2024)
Os-Ni_4Mo/MoO_2/NF	1 M KOH + seawater	500	2500	-	-	~25 ^oC	3	*Adv. Mater.* **36**, 2408982 (2024)
$Ni(OH)_2$–PA–Fe/NF	1 M KOH + seawater	1000	1200	-	-	-	3	*Green Chem.* **27**, 464–472 (2025)
NFB-LDH/Ni mesh	1 M NaOH + seawater + 0.05 M Na_2SO_4	400	10000	-	1	~25 ^oC	2	*Adv. Mater.* **36**, 2411302 (2024)
$LiFePO_4$($Ni(OH)_2$/L-LFP)	1 M KOH + seawater	100	600	-	-	-	3	*Angew. Chem. Int. Ed.* **63**, e202410396 (2024)
$Ni_3FeN@PO_4^{3-}$/NF	1 M KOH + seawater	1000	2500	-	-	~25 ^oC	3	*Adv. Mater.* **37**, 2415421 (2025)
B, Fe-CoP	1 M KOH + seawater	100	200	-	-	-	3	*Adv. Funct. Mater.* **34**, 2402264 (2024)
Ni-BDC/NH_2-MIL-88B(Fe)	1 M KOH + seawater	360	28	-	-	~25 ^oC	3	*Adv. Funct. Mater.* **34**, 2314611

								(2024)
NiFe-MOF@NiS/NF	1 M KOH + seawater	100	600	-	-	-	3	*Chin. J. Catal.* **61**, 192–204 (2024)
$(FeCoNiMnAl)_3O_4$/NF	1 M KOH + seawater	500	50	-	-	-	3	*Appl. Catal. B Environ. Energy* **349**, 123875 (2024)
NiCoP–Cr_2O_3/NF (intermittent electrolysis)	1 M NaOH + seawater	500	10000	-	4	60 ^oC	2 (AEM)	*Nature* **639**, 360–367 (2025)
CP/NF	1 M KOH	200	40	5.2	1	-	3	*Chem. Eng. J.* **408**, 127331 (2021)
NiFe-P_{Zn}@PNTA	1 M KOH	100	360	-	1	~25 ^oC	3	*Adv. Mater.* **35**, 2209500 (2023)
C–$Ni_{1-x}O$/3DPNi	1 M KOH	850	16	-	-	80 ^oC	2	*Adv. Energy Mater.* **10**, 2002955 (2020)
CPNCP/NF	1 M KOH	200	17	5.2	1	-	3	*ACS Appl. Energy Mater.* **3**, 9769–9784 (2020)

Supplementary Table 2. Comparison of η at 1000 mA cm^{-2} for MnO_2@Co-Pi@CoP/NF with previously reported anodes.

Catalyst	Electrolyte	η (mV)	Ref.
MnO_2@Co-Pi@CoP/NF	1 M KOH + seawater	352	This work
NiFe LDH-CeW/NF	1 M KOH + seawater	387	*Appl. Catal. B* **330**, 122612 (2023)
NiFeO-CeO_2/NF	1 M KOH + seawater	408	*ACS Nano* **17**, 16008−16019 (2023)
S-Ni/Fe(OOH)/NF	1 M KOH + seawater	462	*Energy Environ. Sci.* **13**, 3439–3446 (2020)
NiFe-MOF@Ni_2P/$Ni(OH)_2$/NF	1 M KOH + seawater	394	*J. Colloid Interface Sci.* **643**, 17–25 (2023)
(NiFe)C_2O_4/NF	1 M KOH + seawater	349	*Angew. Chem. Int. Ed.* **63**, e202316522 (2024)
$NiMoS_x$@NiFe-LDH/NF	1 M KOH + seawater	350	*Inorg. Chem. Front.* **10**, 2766–2775 (2023)
CrO_4^{2-}-NiFe LDH/Cr_2O_3/NF	1 M KOH + seawater	323	*Nat. Commun.* **15**, 6624 (2024)
Ni_2P-Fe_2P/NF	1 M KOH + seawater	431	*Adv. Funct. Mater.* **31**, 2006484 (2021)
NiCoS/NF	1 M KOH + seawater	470	*Appl. Catal. B* **291**, 120071 (2021)
MnCo/NiSe/NF	1 M KOH + seawater	460.2	*Appl. Catal. B* **325**, 122355 (2023)
RuMoNi/NF	1 M KOH + seawater	470	*Nat. Commun.* **14**, 3607 (2023)
$P_{4.8}$-NiFe ANs-400/NF	1 M KOH + seawater	355	*Appl. Catal. B* **342**, 123376 (2024)
F-$FeCoP_v$@IF	1 M KOH + seawater	370	*Appl. Catal. B* **328**, 122487 (2023)
Ag/NiFe LDH/NF	1 M KOH + seawater	303	*Nano Energy* **98**, 107212 (2022)

$Fe_2P/Ni_{1.5}Co_{1.5}N/Ni_2P/NF$	1 M KOH + seawater	340	*ACS Nano* **17**, 1681−1692 (2023)
B-Co_2Fe LDH/NF	1 M KOH + seawater	415	*Nano Energy* **83**, 105838 (2021)
CoFeOF/NF	1 M KOH + seawater	380	*Chem. Eng. J.* **480**, 146545 (2024)
Mn-FeP_v/NF	1 M KOH + seawater	395	*Small* **20**, 2308613 (2024)
TS-NiFe LDH/NF	1 M KOH + seawater	412	*Small* **20**, 2311431 (2024)
RuNi-Fe_2O_3/NF	1 M KOH + seawater	497	*Chin. J. Catal.* **43**, 2202–2211 (2022)
Ru-FeP_4/NF	1 M KOH + seawater	520	*Appl. Catal. B* **319**, 121950 (2022)
S-Cu_2O-CuO	1 M KOH + seawater	540	*Catal. Today* **400–401**, 14–25 (2022)

Supplementary Table 3. Comparison of lifespan at different *j* for MnO_2@Co-Pi@CoP/NF with previously reported anodes.

Catalyst	Electrolyte	*j* (mA cm^{-2})	Time (h)	Ref.
MnO_2@Co-Pi@CoP/NF	1 M KOH + seawater	1000<br>2000	3000<br>800	This work
NiMoN@NiFeN/NF	1 M KOH + seawater	500	24	*Nat. Commun.* **10**, 5106 (2019)
Fe-NiSOH/NF	1 M KOH + seawater	500	900	*Energy Environ. Sci.* **15**, 4647–4658 (2022)
BZ-NiFe-LDH/NF	1 M KOH + seawater	500	100	*Nano Res. Energy* **1**, e9120028 (2022)
S-Ni/Fe(OOH)/NF	1 M KOH + seawater	100	100	*Energy Environ. Sci.* **13**, 3439–3446 (2020)
(NiFe)C_2O_4/NF	1 M KOH + seawater	1000	600	*Angew. Chem. Int. Ed.* **63**, e202316522 (2024)
CoP_x@FeOOH/NF	1 M KOH + seawater	500	80	*Appl. Catal. B* **294**, 120256 (2021)
CrO_4^{2-}-NiFe LDH/Cr_2O_3/NF	1 M KOH + seawater	1000	1000	*Nat. Commun.* **15**, 6624 (2024)
Ni_2P-Fe_2P/NF	1 M KOH + seawater	500	23	*Adv. Funct. Mater.* **31**, 2006484 (2021)
NiCoS/NF	1 M KOH + seawater	100	100	*Appl. Catal. B* **291**, 120071 (2021)
$NiMoS_x$@NiFe-LDH/NF	1 M KOH + seawater	500	500	*Inorg. Chem. Front.* **10**, 2766–2775 (2023)
NiFe/NiS_x/NF	1 M KOH + seawater	400	1000	*Proc. Natl. Acad. Sci.* **116**, 6624–6629 (2019)
NiIr-LDH/NF	1 M KOH + seawater	500	650	*J. Am. Chem. Soc.* **144**, 9254–9263 (2022)
RuMoNi/NF	1 M KOH + seawater	500	3000	*Nat. Commun.* **14**, 3607 (2023)
MoO_3@CoO/CC	1 M KOH + seawater	600	1000	*Nat. Commun.* **15**, 2481 (2024)

$P_{4.8}$-NiFe ANs-400/NF	1 M KOH + seawater	100	100	*Appl. Catal. B* **342**, 123376 (2024)
NiCoP foam	1 M KOH + seawater	1000	300	*J. Mater. Chem. A* **12**, 2680–2684 (2024)
Ag/NiFe LDH/NF	1 M KOH + seawater	1000	1000	*Nano Energy* **98**, 107212 (2022)
$Fe_2P/Ni_{1.5}Co_{1.5}N/Ni_2P$/NF	1 M KOH + seawater	100	40	*ACS Nano* **17**, 1681–1692 (2023)
NiFeBa-LDH/NF	1 M NaOH + seawater + 0.05 Na_2SO_4	400	10000	*Adv. Mater.* **36**, 2411302 (2024)
$Ni_3FeN@PO_4^{3-}$/NF	1 M KOH + seawater	1000	2500	*Adv. Mater.* **37**, 2415421 (2025)
NiCoP–Cr_2O_3/NF	1 M NaOH + seawater	500	10000 (intermittent electrolysis)	*Nature* **639**, 360–367 (2025)

Supplementary Table 4. Mechanical characteristic parameters of powder MnO_2@Co-Pi@CoP and powder CoP.

Catalyst	Density (g cm^{-3})	Young modulus (Mpa)	Poisson ratio
MnO_2@Co-Pi@CoP	2.4737	517	0.3
CoP	2.22844	515	0.3
